# Minimally invasive transforaminal lumbar interbody fusion versus oblique lateral interbody fusion for lumbar degenerative disease: a meta-analysis

**DOI:** 10.1186/s12891-021-04687-7

**Published:** 2021-09-18

**Authors:** Qing-Yi Zhang, Jie Tan, Kai Huang, Hui-Qi Xie

**Affiliations:** Laboratory of Stem Cell and Tissue Engineering, Orthopaedic Research Institute, Department of Orthopaedics, West China Hospital, Sichuan University, Keyuan fourth Road, Gaopeng Avenue, Chengdu, Sichuan 610041 People’s Republic of China

**Keywords:** Minimally invasive transforaminal lumbar interbody fusion (MIS-TLIF), Oblique lateral interbody fusion (OLIF), Degenerative lumbar diseases

## Abstract

**Background:**

Minimally invasive transforaminal lumbar interbody fusion (MIS-TLIF) and oblique lateral interbody fusion (OLIF) are widely used in the treatment of lumbar degenerative diseases. In the present study, a meta-analysis was conducted to compare the clinical and radiographic efficacy of these two procedures.

**Methods:**

A systematic literature review was performed, and the quality of retrieved studies was evaluated with the Newcastle-Ottawa Scale (NOS). Clinical outcomes, including operation time, intraoperative blood loss, improvement in Visual Analogue Scale (VAS), improvement in Oswestry Disability Index (ODI), Japanese Orthopaedic Association Back Pain Evaluation Questionnaire (JOABPEQ) effectiveness rate and complications, in addition to radiographic outcomes, including restoration of disc height, disc angle, overall lumbar lordosis, fusion rate and subsidence, were extracted and input into a fixed or random effect model to compare the efficacy of MIS-TLIF and OLIF.

**Results:**

Seven qualified studies were included. Clinically, OLIF resulted in less intraoperative blood loss and shorter operation time than MIS-TLIF. Improvement of VAS for leg pain was more obvious in the OLIF group (*P* < 0.0001), whereas improvement of VAS for back pain (*P* = 0.08) and ODI (*P* = 0.98) as well as JOABPEQ effectiveness rate (*P* = 0.18) were similar in the two groups. Radiographically, OLIF was more effective in restoring disc height (*P* = 0.01) and equivalent in improving the disc angle (*P* = 0.18) and lumbar lordosis (*P* = 0.48) compared with MIS-TLIF. The fusion rate (*P* = 0.11) was similar in both groups, while the subsidence was more severe in the MIS-TLIF group (*P* < 0.00001).

**Conclusions:**

The above evidence suggests that OLIF is associated with a shorter operation time (with supplementary fixation in the prone position) and less intraoperative blood loss than MIS-TLIF and can lead to better leg pain alleviation, disc height restoration and subsidence resistance. No differences regarding back pain relief, functional recovery, complications, disc angle restoration, lumbar lordosis restoration and fusion rate were found. However, due to the limited number of studies, our results should be confirmed with high-level studies to fully compare the therapeutic efficacy of MIS-TLIF and OLIF.

**Trial registration:**

PROSPERO ID: CRD42020201903.

**Supplementary Information:**

The online version contains supplementary material available at 10.1186/s12891-021-04687-7.

## Background

Lumbar interbody fusion is effective in enhancing arthrodesis, inducing lumbar lordosis and decompressing neural elements [1–3]; therefore, it has been widely used as the gold-standard treatment for a variety of end-stage lumbar degenerative disorders, including stenosis, disc disease, spondylolisthesis and deformity [4]. Studies have validated the therapeutic effect of conventional open posterior or anterolateral surgeries [5, 6]. However, due to significant iatrogenic injury and approach-related morbidity, these surgeries have been gradually replaced by newly developed minimally invasive procedures [7, 8].

As an alternative to conventional posterior-approach surgery [9], minimally invasive transforaminal lumbar interbody fusion (MIS-TLIF) not only reduces iatrogenic soft tissue injury during spinal exposure [10, 11] but also minimizes the retraction of the dural sac and nerve root through its transforaminal corridor [12, 13]. In contrast, oblique lateral interbody fusion (OLIF) is a revised type of anterolateral-approach technique that uses the retroperitoneal corridor to access the intervertebral space and decompresses the neural structure in an indirect manner [14, 15]. OLIF effectively avoids hypogastric or lumbar plexus injuries, and it keeps the psoas intact. In addition, OLIF preserves the posterior structures [16, 17]. To date, both MIS-TLIF and OLIF are steadily gaining acceptance as the choice of fusion methods as their clinical and radiographic efficacy has been demonstrated by a growing body of evidence [9, 18–23].

Nevertheless, due to the distinct surgical approach and fusion strategy of MIS-TLIF and OLIF, it remains to be determined which procedure leads to better outcomes. To our knowledge, only a few studies have compared them directly, and convincing evidence is still lacking. In this context, a meta-analysis was performed to compare the clinical and radiographic outcomes of MIS-TLIF and OLIF to provide an evidence-based reference for clinicians.

## Methods

### Research strategy and selection criteria

A systematic literature review was performed by following the Preferred Reporting Items for Systematic Reviews and Meta-Analysis (PRISMA) guidelines [24] (checklist is shown in Supplementary Table 1). Relevant studies (by January 17, 2021) in PubMed, Embase, Cochrane Library, China National Knowledge Internet (CNKI), Wanfang Data and Chongqing VIP database (CQVIP) were retrieved to identify studies comparing MIS-TLIF and OLIF for the treatment of lumbar degenerative disorders. The following search terms were used: ((((oblique) OR (retroperitoneal)) OR (OLIF)) AND ((transforaminal) OR (TLIF))) AND (((((((((Spinal Fusion [MeSH Terms]) OR (Spinal Fusion [Title/Abstract])) OR (Fusion, Spinal [Title/Abstract])) OR (Fusions, Spinal [Title/Abstract])) OR (Spinal Fusions [Title/Abstract])) OR (Spondylodesis [Title/Abstract])) OR (Spondylodeses [Title/Abstract])) OR (Spondylosyndesis [Title/Abstract])) OR (Spondylosyndeses [Title/Abstract])). Relevant studies from references were also checked to broaden our search. The inclusion criteria were as follows: (1) prospective or retrospective studies that compared single-level MIS-TLIF and OLIF for the treatment of lumbar degenerative disorders; (2) studies that provided information in regard to clinical and radiographic efficacy (including at least one of the following: operation time, intraoperative blood loss, Visual Analogue Scale (VAS), Oswestry Disability Index (ODI), Japanese Orthopaedic Association Back Pain Evaluation Questionnaire (JOABPEQ) effectiveness rate, complications, disc height, disc angle, lumbar lordosis, fusion rate and subsidence); (3) studies with a mean follow-up of more than 6 months; and (4) studies published in English or Chinese. Studies that met the following criteria were excluded: (1) noncomparative study; (2) combination of both techniques in the same treatment; and (3) case report, letter, comment, review and conference papers.

### Data extraction and quality assessment

Titles, abstracts and, if necessary, the full text of the eligible studies were independently reviewed by two investigators. The risk of bias was assessed using the Newcastle-Ottawa Scale (NOS), in which each study was assessed in terms of selection, comparability and exposure/outcome. Studies gaining with more than five “stars” were included in the analysis. The following data were extracted by the same investigators: publishing information (first author, publication year, location of the study, period of the study and design type), demographic characteristics (age, sex, diagnosis and surgical procedures) and outcomes of interest (blood loss, operation time, VAS, ODI, JOABPEQ effectiveness rate, complications, disc height, disc angle, overall lumbar lordosis, fusion rate and subsidence). Disagreement was resolved by discussion with all authors.

### Statistical analysis

All data were analysed within Review Manager (RevMan version 5.3, Cochrane Collaboration, Oxford, UK). Continuous variables were analysed as standard mean differences (SMDs), and dichotomous variables were analysed by odds ratios (ORs). *χ*^*2*^ and *I*^*2*^ tests were performed to evaluate heterogeneity where *P* > 0.1 or *I*^*2*^ < 50 % was considered homogeneous among studies. Under such circumstances, a fixed-effects model was used, or a random-effects model was constructed. *P* ≤ 0.05 was considered to be statistically significant. Publication bias was assessed using a visual funnel plot. Besides, sensitivity analysis was performed by eliminating one study at a time to assess the resolution of heterogeneity [25].

## Results

### Study selection

**Fig. 1:**
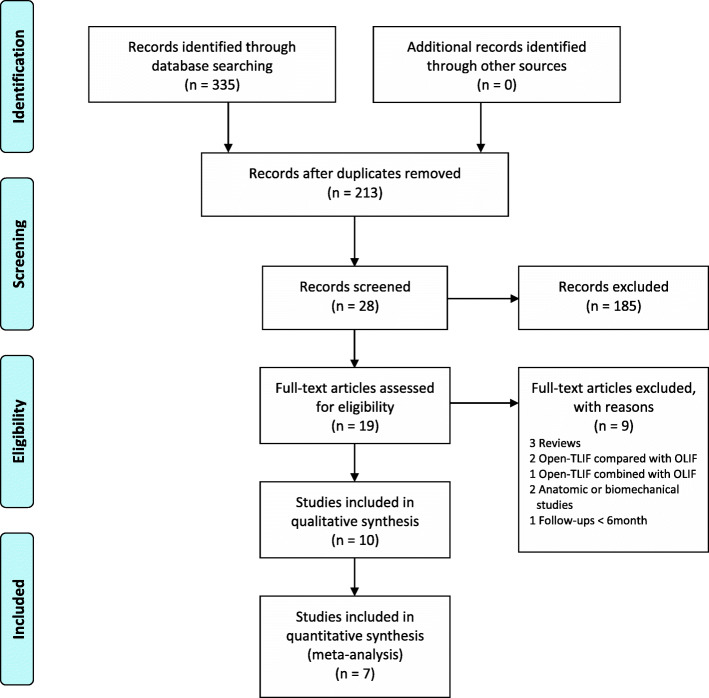
Study selection flow diagram for the meta-analysis

A total of 335 studies were initially identified, including 171 published in English and 164 in Chinese. In total, 122 studies were removed due to duplication, and 185 studies were excluded after screening the titles and abstracts. The remaining 19 studies were fully reviewed. Ultimately, 7 studies met the criteria and were included in the meta-analysis [26–32]. The detailed selection process is summarized in Fig. 1.

### Characteristics of studies

Seven retrospective cohort studies published between 2012 and 2020 were included [26–32], and all of which were considered to be of moderate-to-high quality according to NOS assessment. A total of 503 patients who underwent single-level interbody fusion (245 in the MIS-TLIF group and 258 in the OLIF group with ages of 64.93 ± 13.04 years and 67.17 ± 11.79 years, respectively) were identified. Spondylolisthesis and spinal stenosis were the most common diagnoses for surgical intervention. Other indications included disc herniation and spinal deformity. The mean follow-ups were 29.2 ± 20.4 months and 23.1 ± 11.8 months in the MIS-TLIF and OLIF groups, respectively. Basic characteristics are summarized in Table 1.

**Table 1:** Characteristics of studies included in the meta-analysis

Study, year	Location	Study period	Study design	Diagnosis	Simple size (Male/Female)		Mean age (year)		Follow-up (month)		NOS
					MIS-TLIF	OLIF	MIS-TLIF	OLIF	MIS-TLIF	OLIF	
Lin, [26], 2018	South Korea	2012-2017	Retrospective cohort study (matched-pair)	spondylolisthesis and spinal stenosis	8/17	8/17	64 ± 10.5	64 ± 7.4	40 ± 17.1	29 ± 10.5	6★
Chen [27], 2018	China	2016-2017	Retrospective cohort study	Spondylolisthesis, spinal stenosis, disc herniation and scoliosis	6/7	6/12	66 ± 12	66 ± 11	11 ± 3.3	11 ± 3.3	6★
Qiu, [28], 2020	China	2018	Retrospective cohort study (matched-pair)	Spondylolisthesis	13/7	15/5	51.7 ± 8.7	50.3 ± 8.8	14.1 ± 2.8	13.5 ± 2.3	7★
Sheng [29], 2020	China	2014-2018	Retrospective cohort study	Spinal stenosis	23/28	5/27	60.6 ± 12.4	65.3 ± 8.9	≥12	≥12	6★
Kokie [30], 2020	Japan	2013-2017	Retrospective cohort study	Spondylolisthesis	18/30	20/18	70.1 ± 11.5	2.1 ± 11.4	22.5 ± 12.8	18.1 ± 8.5	6★
Kotani [31], 2020-1	Japan	2013-2018	Retrospective cohort study	Spondylolisthesis	17/33	46/46	70.0 ± 11.2	72.0 ± 9.9	57.2 ± 7.2	31 ± 11.5	7★
Kotani [32], 2020-2	Japan	2012-2018	Retrospective cohort study	Spinal stenosis, spondylolisthesis, disc herniation and pseudarthrosis	25/13	15/18	64.7 ± 15.3	63.1 ± 12.5	31.0 ± 20.0	25.4 ± 7.6	7★

*MIS-TLIF* minimally invasive transforaminal interbody fusion, *OLIF* oblique lateral interbody fusion, *NOS* Newcastle-Ottawa Scale

### Clinical outcomes

**Fig. 2:**
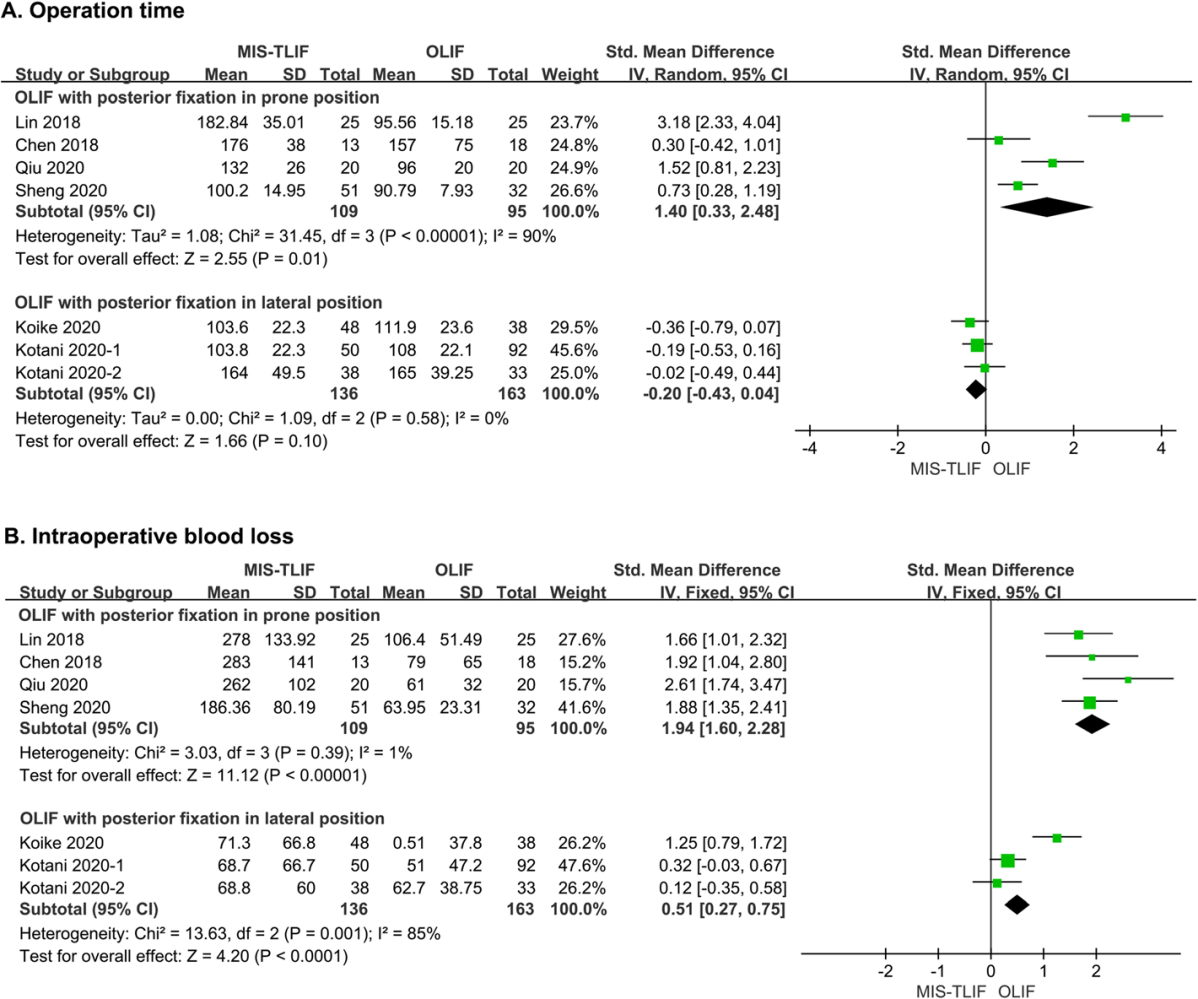
Forest plots comparing (**A**) operation time and  (**B**) intraoperative blood loss between MIS-TLIF and OLIF. MIS-TLIF: minimally invasive transforaminal interbody fusion, OLIF: oblique lateral interbody fusion

All seven studies recorded operation time and intraoperative blood loss [26–32]. However, due to the heterogeneity caused by different fixation procedures in OLIF, subgroup analysis was performed. Specifically, in four studies, OLIF with posterior fixation in the prone position required less operation time than MIS-TLIF (SMD = 1.40, 95 % CI = 0.33 ~ 2.48, *P* = 0.01, *I*^*2*^ = 90 %) [26–29]. In the other three studies in which supplementary fixation was performed in the lateral position (as an attempt to further reduce operation time), the advantage of OLIF diminished (SMD = 0.-20, 95 % CI = -0.43 ~ 0.04, *P* = 0.10, *I*^*2*^ = 0 %; Fig. 2A) [30–32]. Similarly, blood loss was reduced in OLIF with fixation in the prone position compared with MIS-TLIF (SMD = 1.94, 95 % CI = 1.60 ~ 2.28, *P* < 0.00001, *I*^*2*^ = 1 %) [26–29]. In contrast, this superiority of OLIF with fixation in the lateral position remained even though it tended to be attenuated (SMD = 0.51, 95 % CI = 0.27 ~ 0.75, *P* < 0.001, *I*^*2*^ = 0 %; Fig. 2B) [30–32].

**Fig. 3:**
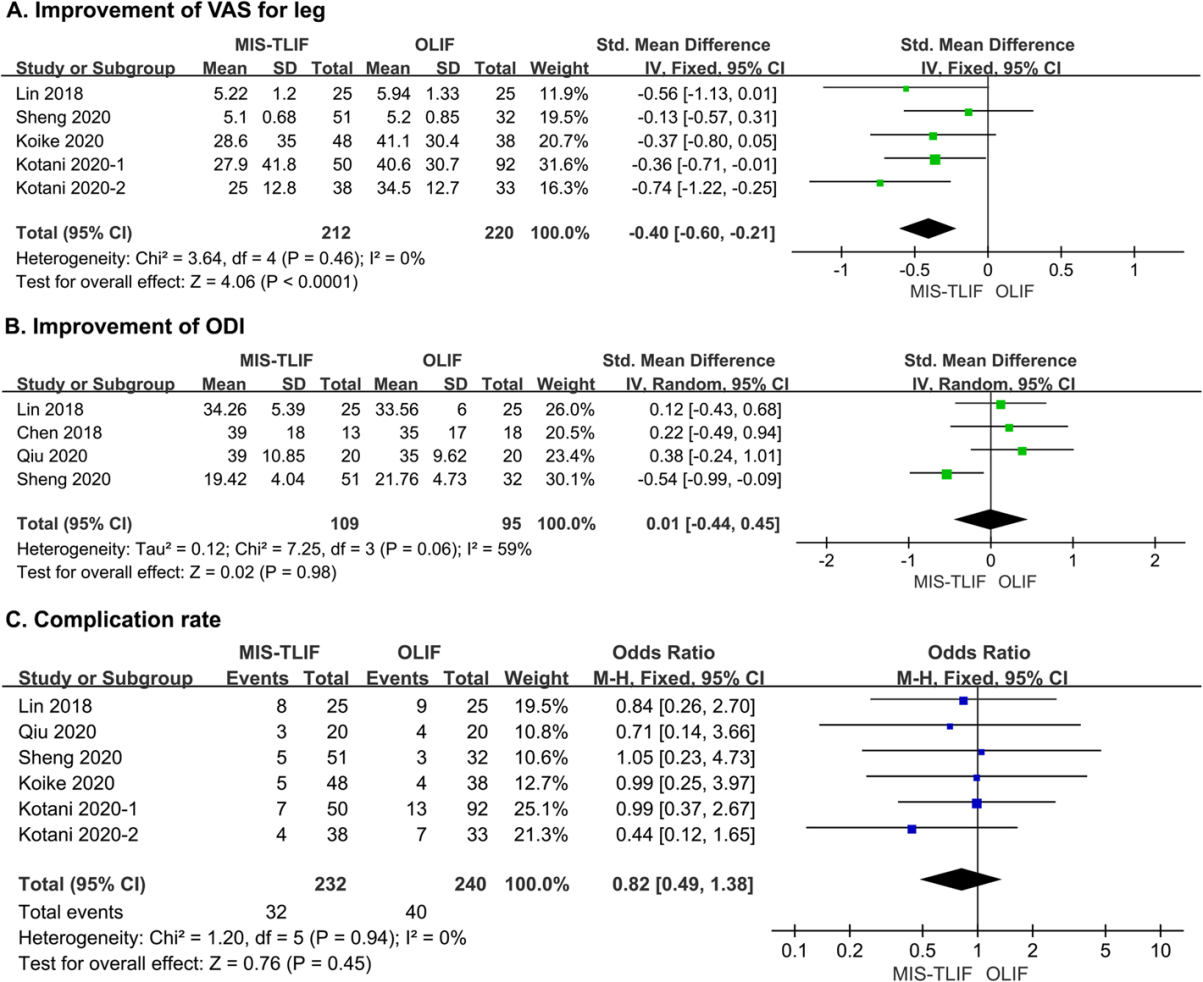
Forest plots comparing (**A**) improvements of VAS for leg pain, **B** improvement of ODI and  **C** complication rate between MIS-TLIF and OLIF. MIS-TLIF: minimally invasive transforaminal fusion, OLIF: oblique lateral interbody fusion, VAS: visual analogue scale; ODI: Oswestry Disability Index

VAS scores for the back and leg were compared in five studies [26, 29–32]. Intriguingly, leg pain showed more remarkable relief after OLIF than after MIS-TLIF (SMD = -0.40, 95 % CI = -0.60 ~ -0.21, *P* < 0.0001, *I*^*2*^ = 0 %; Fig. 3A). For back pain alleviation, the advantage of OLIF was marginal (SMD = -0.18, 95 % CI = -0.37 ~ 0.02, *P* = 0.08, *I*^*2*^ = 0 %; Supplementary Fig. 1 A). For functional recovery measured by ODI, pooled analysis of four studies suggested that the efficacy of the two methods was similar (SMD = 0.01, 95 % = − 0.44 ~ 0.45, *P* = 0.98, *I*^*2*^ = 59 %; Fig. 3B) [26–29]. JOABPEQ effectiveness rates calculated in three studies also showed no significant differences in postoperative lumbar function, walking ability, social life or mental health between the two procedures. Only terms related to pain relief were consistent with the VAS score and favoured OLIF (Supplementary Fig. 1B) [30–32].

Complications were recorded in six studies [26, 28–32]. A total of 32 patients in the MIS-TLIF group and 39 patients in the OLIF group developed postoperative complications. Pooled analysis showed no significant difference in the overall incidence between MIS-TLIF and OLIF (OR = 0.82, 95 % CI = 0.49 ~ 1.38, *P* = 0.45, *I*^*2*^ = 0 %; Fig. 3C). Both approach-related and unrelated complications are summarized in Table 2, and no significant difference was detected (Supplementary Fig. 2A - B).

**Table 2:** Summary of complications of included studies

Complications, N (%)	MIS-TLIF (***N*** = 232)	OLIF (***N*** = 240)
**Approach-related**		
Dural tear and root injury	4 (1.7 %)	0 (0 %)
Ileus	0 (0 %)	1 (0.4 %)
Leg pain	2 (0.9 %)	3 (1.3 %)
Thigh numbness	3 (0.9 %)	3 (1.3 %)
Hip flexion weakness	2 (1.3 %)	4 (1.7 %)
Segmental artery injury	0 (0 %)	1 (0.4 %)
Sympathetic chain injury	0 (0 %)	3 (1.3 %)
**Approach-unrelated**		
Adjacent segment disease	9 (3.9 %)	12 (5 %)
Edema	0 (0 %)	3 (1.3 %)
Deep venous thrombosis	1 (0.4 %)	0 (0 %)
Infection	1 (0.4 %)	0 (0 %)
Late multiple sclerosis	0 (0 %)	1 (0.4 %)
Pseudarthrosis	6 (2.6 %)	4 (1.7 %)
Pulmonary thromboembolism	1 (0.4 %)	0 (0 %)
Screw and cage incidence	2 (0.9 %)	4 (1.7 %)
Thrombocytopenia	1 (0.4 %)	0 (0 %)
**Total**	32 (13.8 %)	39 (16.3 %)

*MIS-TLIF* minimally invasive transforaminal interbody fusion, *OLIF* oblique lateral interbody fusion

### Radiographic outcomes

**Fig. 4:**
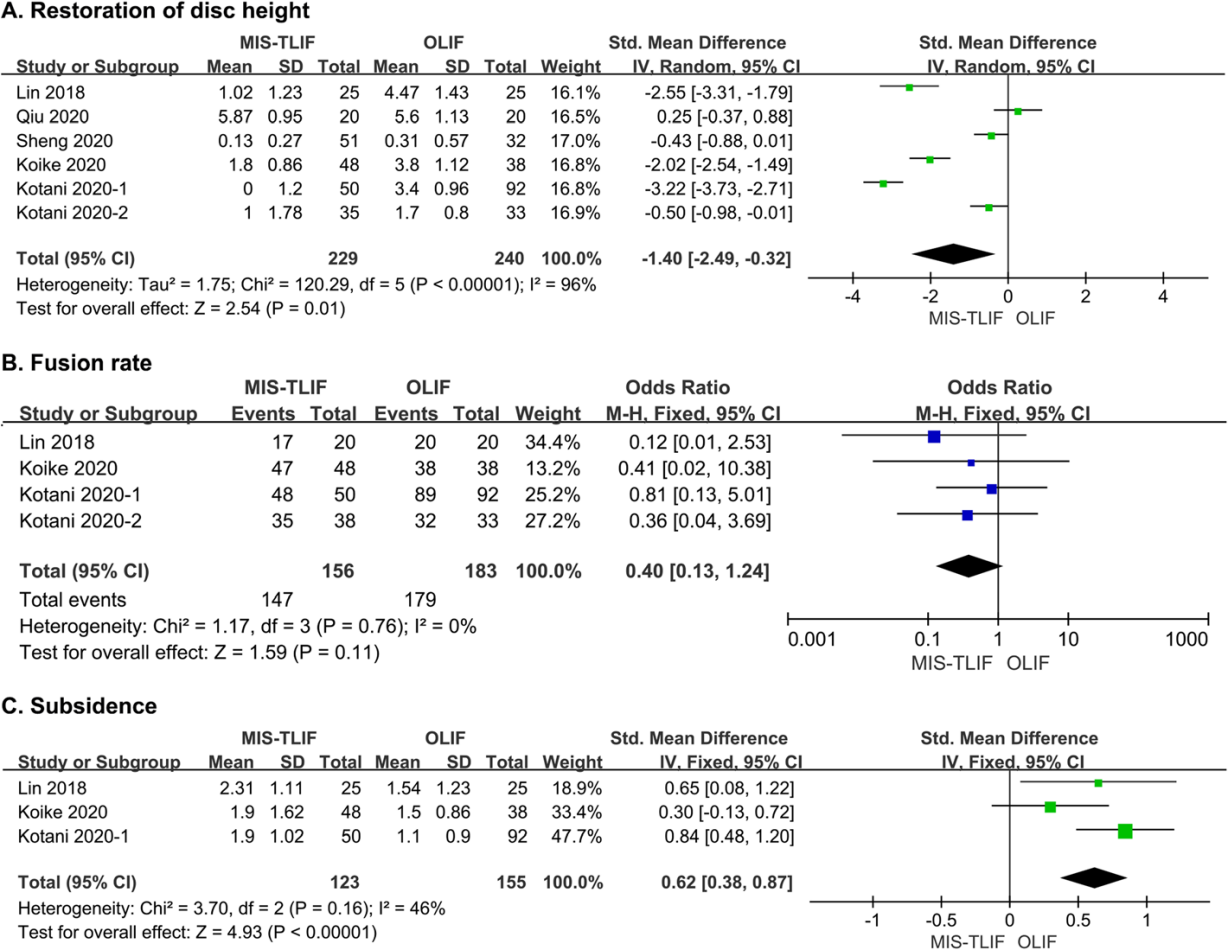
Forest plots comparing (**A**) restoration of disc height, **B** fusion rate and **C** subsidence between MIS-TLIF and OLIF. MIS-TLIF: minimally invasive transforaminal interbody fusion, OLIF: oblique lateral interbody fusion

Disc height was measured in six studies [26, 28–32]. Pooled analysis showed that OLIF resulted in better restoration of disc height at the last follow-up compared with MIS-TLIF (SMD = -1.40, 95 % CI = -2.49 ~ -0.32, *P* = 0.01, *I*^*2*^ = 96 %; Fig. 4A). The disc angle was measured in six studies, including five performed at L1-5 and one at L5-S1 [26–29, 31, 32]. Considering the heterogeneity, we excluded the latter from the pooled analysis, which indicated that OLIF had a marginal advantage in the restoration of the disc angle (SMD = -0.15, 95 % CI = -0.37 ~ 0.07, *P* = 0.18, *I*^*2*^ = 0 %; Supplementary Fig. 3A) [26–30, 32]. Surprisingly, OLIF at the L5-S1 level attained more apparent improvement in the disc angle compared to MIS-TLIF (5.6 ± 3.6 in OLIF vs. 0.5 ± 3.3 in MIS-TLIF) [31]. In contrast, pooled analysis of four studies revealed no difference in the improvement of overall lumbar lordosis between the two methods (SMD = -0.11, 95 % CI = -0.41 ~ 0.19, *P* = 0.48, *I*^*2*^ = 0 %; Supplementary Fig. 3B) [26–29].

Four studies provided detailed data on the fusion rate [26, 30–32]. OLIF had a higher fusion rate than MIS-TLIF, but the difference was not statistically significant (OR = 0.40, 95 % CI = 0.13 ~ 1.24, *P* = 0.11, *I*^*2*^ = 0 %; Fig. 4B). Subsidence during the follow-up was recorded in three studies [26, 31, 32]. Pooled analysis indicated that OLIF incurred significantly less disc height loss than MIS-TLIF (SMD = 0.62, 95 % CI = 0.38 ~ 0.87, *P* < 0.00001, *I*^*2*^ = 46 %; Fig. 4C).

### Sensitivity analysis

Funnel plots were constructed to assess publication bias, and the results were largely symmetric, suggesting acceptable publication bias in our analysis (Supplementary Fig. 4). Sensitivity analysis was also performed by randomly excluding one study at a time. All results were confirmed to be stable with *P* ≤ 0.05 after removing any of the included studies.

## Discussion

In recent decades, both MIS-TLIF and OLIF have become the most widely used minimally invasive techniques in the treatment of lumbar degenerative diseases, but their relative efficacy has yet to be determined. By systematically comparing their outcomes in single-level interbody fusion, our analysis suggested that OLIF remarkably reduces the operation time (with supplementary posterior fixation in the prone position) and intraoperative blood loss compared with MIS-TLIF. This improvement may be attributed to the delicate retroperitoneal approach in OLIF, where the surgeons access the intervertebral spaces through the natural corridor between arteries and psoas muscles by simple blunt dissection instead of successive paraspinal muscle dilation and facetectomy as in MIS-TLIF [23, 33]. Moreover, direct decompression is avoided in OLIF, which largely eliminates the additional time occupied by microscopy and other specific complex ancillaries for neuroprotection or monitoring [14, 34]. In three Japanese studies, surgeons attempted to accomplish posterior fixation in the same lateral position to reduce the additional operation time caused by position changes in OLIF [30–32]. Unexpectedly, the operation time did not decrease and was similar to that of MIS-TLIF, which might be due to the limited working space and difficulty in fluoroscopy at this position. In the present study, we only analysed single-level interbody fusion. In fact, OLIF achieves multilevel fusion through the same corridor, while separate channels are required in MIS-TLIF. Theoretically, we assume that the superiority of OLIF in operation time and intraoperative blood loss may further expand with the increase in the number of fusion segments [35, 36].

Despite being less invasive, it remains unclear whether OLIF attains similar clinical effects to MIS-TLIF. Unlike MIS-TLIF, OLIF only achieves neural decompression indirectly through obliteration of the bulging disc and ligamentotaxis [37]. In fact, our pooled analysis indicated that OLIF may be even more effective in alleviating leg pain, which agreed with the imaging finding that a significantly increased cross-sectional area of the foramen is observed in patients undergoing OLIF rather than MIS-TLIF, suggesting a better performance of OLIF in nerve decompression [26]. Additionally, as OLIF does not open the spinal canal, stimulation to nerve roots is avoided, which may further alleviate leg symptoms after surgery [38]. Although without statistical significance, OLIF still showed an advantage in reducing pain in the back. Some surgeons have proposed that this advantage may be attributed to the better protection of lumbar fascia and paravertebral muscles in OLIF [31]. In terms of functional recovery, improvement of the ODI scores was similar between MIS-TLIF and OLIF. A similar efficacy was also confirmed by the JOABPEQ effectiveness rate regarding lumbar function, walking ability, social life and mental health. Only term associated with pain was in line with the VAS score, which favoured OLIF.

With respect to complications, the overall complication incidence of MIS-TLIF (13.8 %) was slightly less than that of OLIF (16.3 %); however, the difference was statistically nonsignificant. Attributed to the narrow transforaminal corridor, dural tears and nerve root injuries are the most common approach-related complications in MIS-TLIF [11, 39, 40]. In contrast, thigh numbness along with hip flexor weakness are frequent after OLIF, which might be due to damage to psoas nerve branches and continuous psoas traction [41–43]. Usually, most symptoms disappear in three months [44]. Approach-unrelated complications, including deep venous thrombosis, infection and pulmonary thromboembolism, were comparable between MIS-TLIF and OLIF. However, at the same time, some studies have also revealed a shorter bedridden time after OLIF, suggesting that it may be beneficial for the rehabilitation and reduction of bedridden complications [45].

Radiographically, pooled analysis demonstrated that OLIF led to better disc height improvement than MIS-TLIF [26, 42], which may be due to the generation of a significantly large annular window in OLIF for sufficient anterior release and high-profile cage implantation [46, 47]. Due to the obstacles of the dural sac, nerve roots and other posterior appendages, it is almost impossible to insert a large cage through the transforaminal channel [48]. Additionally, the cages used in OLIF may have a favourable angle between the upper and lower surfaces to induce lordosis (up to 12°), while it is almost plain in MIS-TLIF; therefore, restoration is only achieved by compressing the posterior column [9, 21, 49–51]. Theoretically, OLIF can restore more lordosis and overall spinal alignment than MIS-TLIF. However, our pooled analysis only detected a slight edge with no statistical significance after fusion at L1-5 between the two procedures. We assume that such discrepancy may not be fully reflected in a single-level fusion, and with the increase in fusion segments, the advantages of OLIF in lordosis creation may accumulate and tend to be statistically significant [27, 51–55]. Actually, current studies have partly verified our hypothesis. For example, Champagne et al. reported a mean improvement of 5.5 degrees in lumbar lordosis after multi-level OLIF, far more than a mean improvement of -0.84 degrees after multi-level MIS-TLIF or TLIF [51]. He et al. and Chen et al. also revealed better improvement of segmental lordosis and overall lumbar lordosis after multi-level OLIF when compared with multi-level TLIF [27, 52]. However, additional studies focused on MIS-TLIF vs. OLIF are required.

Although pooled analysis found no significant difference between the two methods in terms of fusion rate at the last follow-up, it has been noted that OLIF leads to segmental fusion much more rapidly than MIS-TLIF [26]. Moreover, subsidence was also significantly lower in the OLIF group. As mentioned above, OLIF provides a much larger annual window to the intended level for comprehensive disc space clearance, endplate preparation and placement of large grafts [44]. In our experience, the footprint of cages in OLIF is at least twice the size of that in MIS-TLIF and is wide enough to stand on both sides of the dense peripheral apophyseal bone [56–59]. Biomechanical analysis has also suggested that such constructs may effectively diminish stress peaks and disperse the stress in the endplate cancellous bone equally, which is beneficial for both fusion and subsidence resistance [60–62].

The present study had several limitations. First, the level of evidence was relatively low because the studies were all retrospective cohort studies. Second, only single-level fusion was analysed due to a lack of related studies, but we assumed that for multilevel fusion, the advantages of OLIF may be more obvious. Third, important radiographic parameters, such as spinopelvic parameters and sagittal spinal balance, were still missing. Finally, long-term outcome was unclear. Due to the above reasons, high-quality studies are still required to validate the respective advantages of MIS-TLIF and OLIF.

## Conclusions

Based on our meta-analysis, OLIF incurs a shorter operation time (with posterior supplementary fixation) and less intraoperative blood loss as well as better alleviation of leg pain, disc height restoration and subsidence resistance than MIS-TLIF in short-term follow-ups. The present study noted no differences in back pain relief, functional recovery, complication rate, disc angle improvement, lumbar lordosis improvement and fusion rate. However, due to the limited number of low-quality studies, our results should be critically evaluated, and high-quality studies to compare the therapeutic efficacy of MIS-TLIF and OLIF are still required.

## Supplementary Information

**Additional file 1.**

**Additional file 2.**

**Additional file 3.**

**Additional file 4.**

**Additional file 5.**

## Data Availability

Data used for analysis was retrieved from openly published studies retrieved from PubMed (https://pubmed.ncbi.nlm.nih.gov), Embase (https://www.embase.com), Cochrane Library (https://www.cochranelibrary.com), China National Knowledge Internet (https://www.cnki.net), Wanfang Data (http://www.wanfangdata.com.cn) and Chongqing VIP database (http://www.cqvip.com).

## Abbreviations

MIS-TLIF: Minimally invasive transforaminal interbody fusion
OLIF: Oblique lateral interbody fusion
NOS: Newcastle-Ottawa Scale
VAS: Visual Analogue Scale
ODI: Oswestry Disability Index
JOABPEQ: Japanese Orthopaedic Association Back Pain Evaluation Questionnaire
OR: Odds ratio
SMD: Standard mean difference
