# Supplementary figures and images for: Minimally invasive transforaminal lumbar interbody fusion versus oblique lateral interbody fusion for lumbar degenerative disease: a meta-analysis
Source: BMC Musculoskelet Disord. 2021 Sep 18;22:802. doi: 10.1186/s12891-021-04687-7 (PMC8449429; doi:10.1186/s12891-021-04687-7)

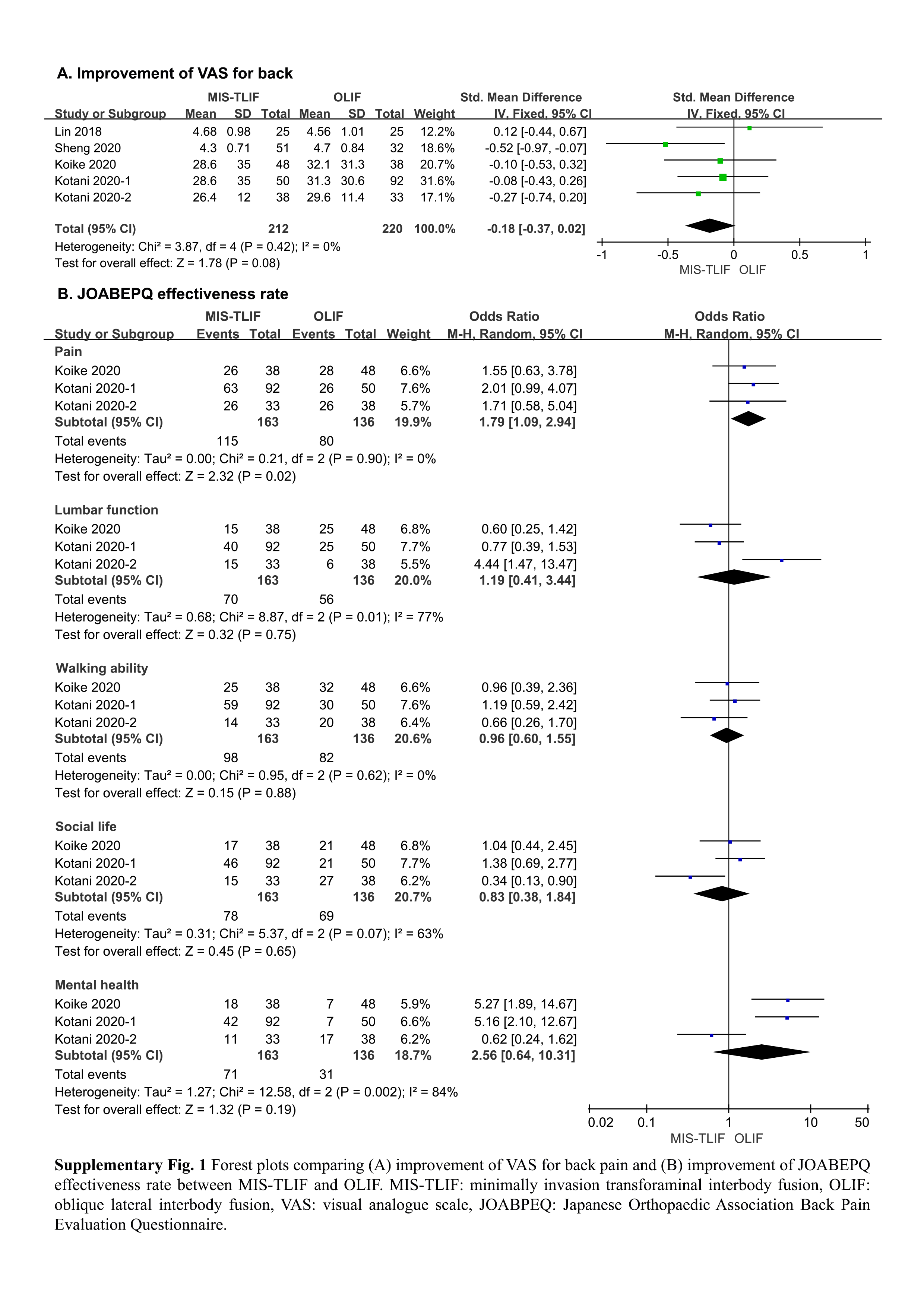

Supplement: Supplementary file 1 — Additional file 1. [file 12891_2021_4687_MOESM1_ESM.tif]

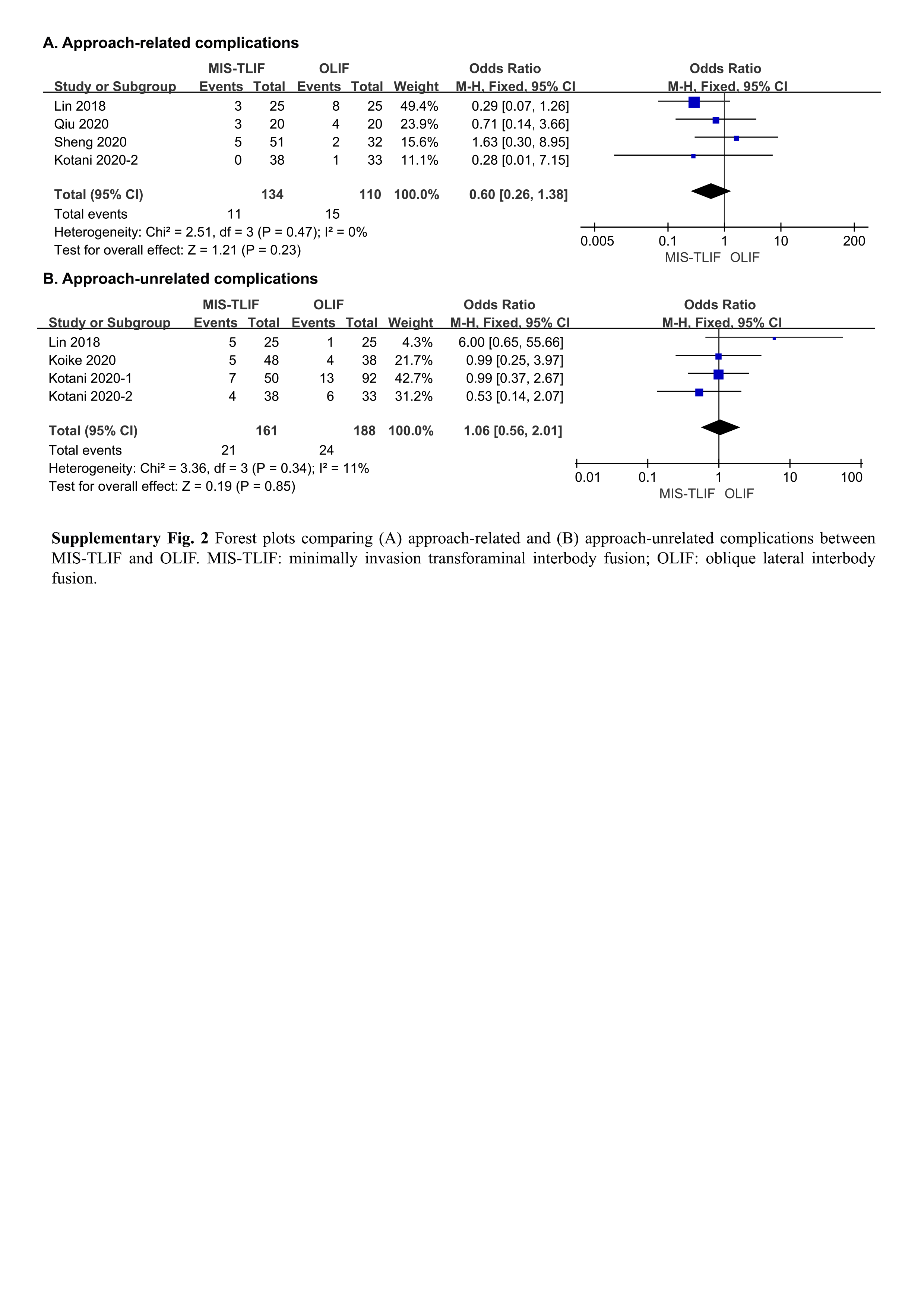

Supplement: Supplementary file 2 — Additional file 2. [file 12891_2021_4687_MOESM2_ESM.tif]

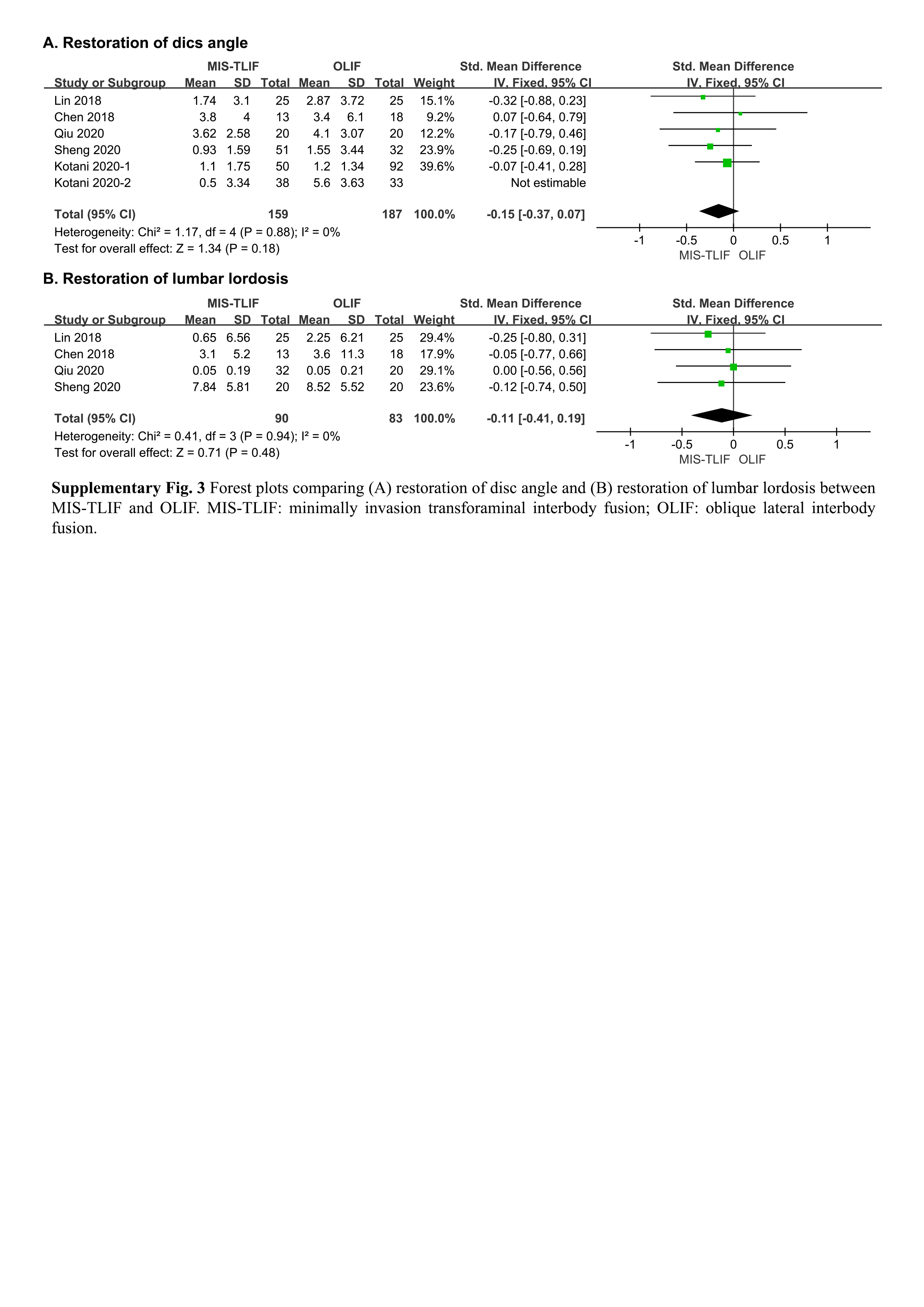

Supplement: Supplementary file 3 — Additional file 3. [file 12891_2021_4687_MOESM3_ESM.tif]

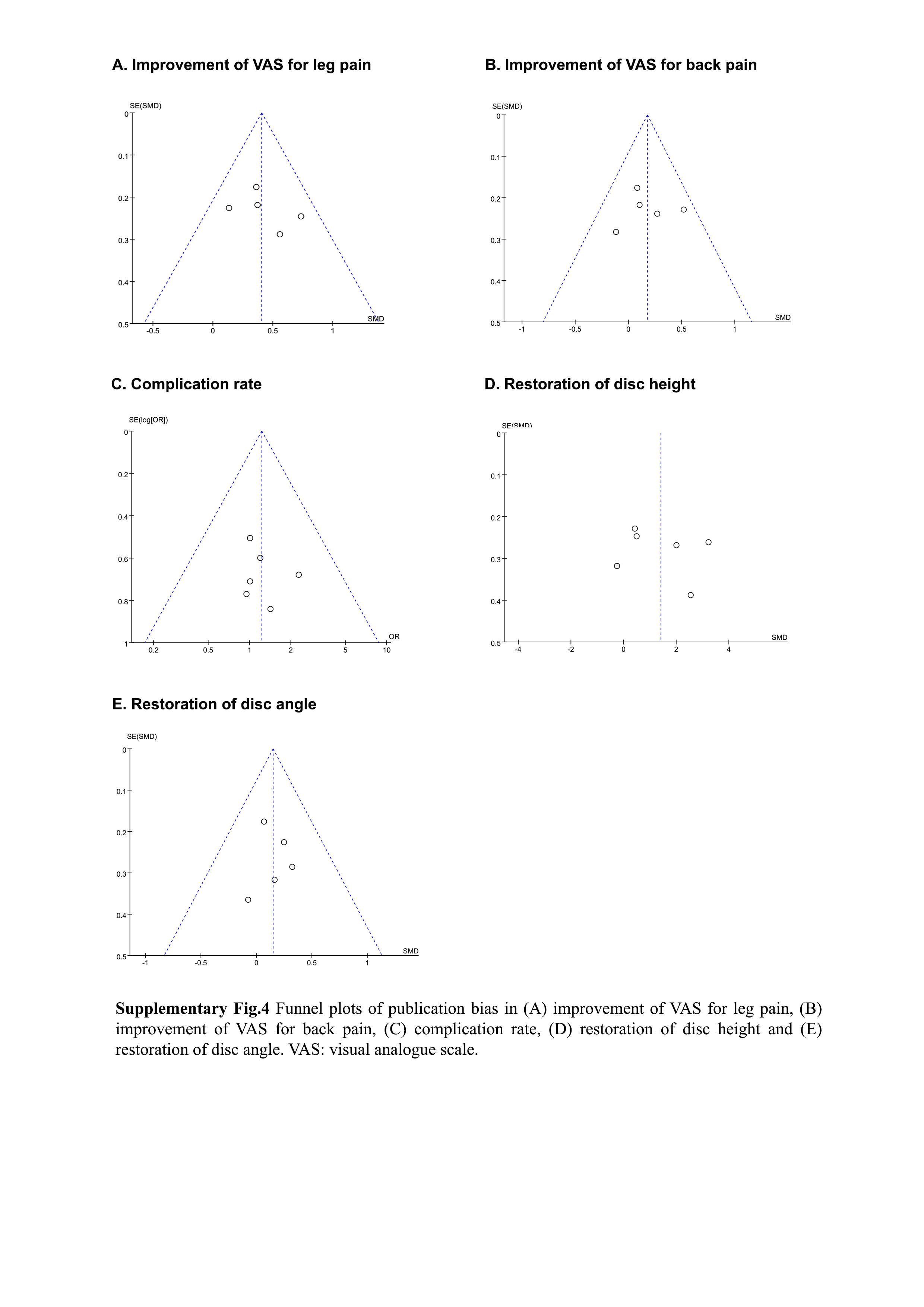

Supplement: Supplementary file 4 — Additional file 4. [file 12891_2021_4687_MOESM4_ESM.tif]
